# Dental Hygiene and Orthodontics: Effect of Ultrasonic Instrumentation on Bonding Efficacy of Different Lingual Orthodontic Brackets

**DOI:** 10.1155/2017/3714651

**Published:** 2017-08-17

**Authors:** Andrea Scribante, Maria Francesca Sfondrini, Vittorio Collesano, Gaia Tovt, Luisa Bernardinelli, Paola Gandini

**Affiliations:** ^1^Unit of Orthodontics and Paediatric Dentistry, Section of Dentistry, Department of Clinical, Surgical, Diagnostic and Paediatric Sciences, University of Pavia, Pavia, Italy; ^2^Unit of Dental Hygiene, Section of Dentistry, Department of Clinical, Surgical, Diagnostic and Paediatric Sciences, University of Pavia, Pavia, Italy; ^3^Section of Statistics, Department of Brain and Behavioural Sciences, University of Pavia, Pavia, Italy

## Abstract

Dental hygienists are often faced with patients wearing lingual orthodontic therapy, as ultrasonic instrumentation (UI) is crucial for oral health. As the application of external forces can lead to premature bonding failure, the aim of this study was to evaluate the effect of UI on shear bond strength (SBS) and on adhesive remnant index (ARI) of different lingual orthodontic brackets. 200 bovine incisors were divided into 10 groups. Four different lingual (STB, Ormco; TTR, Rocky Mountain Orthodontics; Idea, Leone; 2D, Forestadent) and vestibular control (Victory, 3M) brackets were bonded. UI was performed in half of specimens, whereas the other half did not receive any treatment. All groups were tested with a universal testing machine. SBS and ARI values were recorded. Statistical analysis was performed (significance: *P* = 0.05). TTR, Idea, and 2D lingual brackets significantly lowered SBS after UI, whereas for other braces no effect was recorded. Appliances with lower mesh area significantly reduced their adhesion capacity after UI. Moreover groups subjected to UI showed higher ARI scores than controls. UI lowered SBS of lingual appliances of small dimensions so particular care should be posed avoiding prolonged instrumentation around bracket base during plaque removal. Moreover, UI influenced also ARI scores.

## 1. Introduction

Oral hygiene professionals are constantly faced with patients under orthodontic treatment [1] as fixed appliances cause plaque accumulation around bands and brackets [2]. During last years the patients' aesthetic demands are deeply rising, thus increasing the requests for invisible lingual orthodontic therapy [3]. Lingual appliances allow the correction of tooth malocclusions through fixed brackets attached to the lingual tooth surfaces [4]. This technique presents high aesthetic if compared with conventional vestibular orthodontic appliance. During lingual orthodontic therapy, plaque and calculus accumulation has been demonstrated to be equal to [5] or higher [6] than vestibular treatment. Dental hygienists therefore are and will be more and more involved in oral health maintenance of patients with these aesthetic appliances. Oral hygiene protocols for patients during orthodontic treatment include both verbal education and professional treatments with rotating brushes and ultrasonic scalers [7].

Ultrasonic instrumentation (UI) around orthodontic devices can lead to application of unwanted stresses around bracket bases [8]. This is particularly true for lingual appliances that are more complex to reach with scalers.

Accidental application of unwanted forces to orthodontic appliances can cause detachment of brackets. Bond failure can influence treatment duration, total costs, and chair time, so it is undesirable both from the patients and from the clinicians [9].

The effect of UI has been demonstrated to induce no modifications in shear bond strength values of resin cements used for composite restorations [10]. On the other hand, UI around orthodontic bracket bases has been demonstrated to reduce the bond strength values of conventional metallic orthodontic brackets, emphasizing the need for caution during professional oral hygiene procedures in orthodontic patients [8].

The purpose of the present investigation was to evaluate the effect of UI on shear bond strength (SBS) values and adhesive remnant index (ARI) scores of various lingual orthodontic brackets with different bracket base dimensions. The null hypothesis of the present report was that there is no difference in SBS values and ARI scores among the different groups.

## 2. Materials and Methods

The present study has been approved by Unit Institutional Committee Board. Two hundred bovine permanent mandibular incisors were collected. After extraction, teeth were stored in a solution of 0.1% (weight/volume) thymol [11]. Inclusion criteria were no cracks, no caries, no rough or irregular buccal surface, and intact enamel [12]. The teeth were cleaned from soft tissues, embedded in acrylic resin (Leocryl, Leone, Sesto Fiorentino, Italy), and placed in metal rings. Each tooth was oriented so that its bonding surface would be parallel to the force applied during shear test [13] in effort to minimize peel and maximize shear during testing. All specimens have been then assigned to one of 10 groups using random number tables.

**Figure 1:**
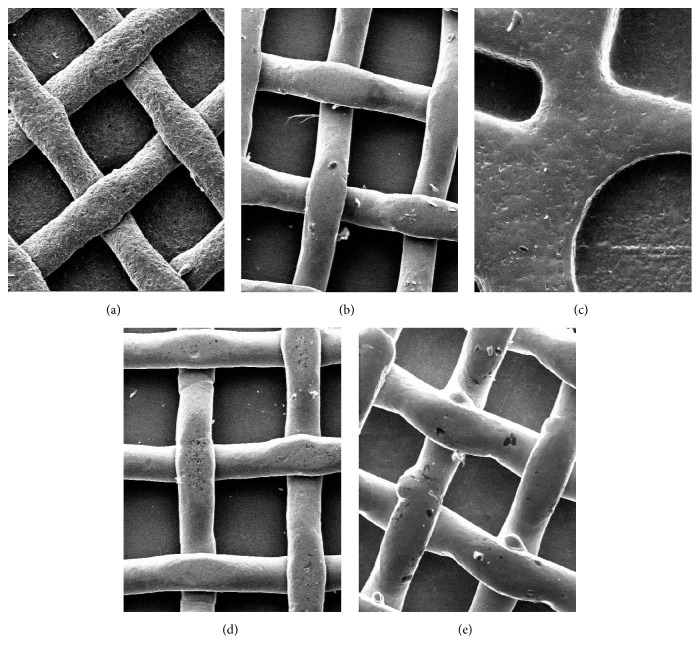
Scanning electron microphotographs of the five different bases of the different brackets tested (a): Victory, 3M; (b): STB, Ormco; (c): TTR, Rocky Mountain Orthodontics; (d): Idea, Leone; (e): 2D, Forestadent.

Before bonding the specimens, 5 microphotographs of the five different bracket bases (Figure 1) were taken using a scanning electron microscope (JSM-6480LV, JEOL Ltd, Tokyo, Japan) to observe differences in bracket bases (×100 magnification). The brackets tested were all for maxillary central incisors.

The facial surface of each incisor was cleansed with a mixture of water and fluoride-free pumice with rubber polishing cup on a low-speed handpiece for 10 s. The enamel surface was rinsed with water to remove any pumice or debris and dried with an oil-free air stream. Teeth were then conditioned with 37% orthophosphoric acid gel (orthophosphoric acid gel, 3M Unitek, Monrovia, California, USA) for 30 seconds, then washed, and dried. Subsequently four different lingual brackets (STB, Ormco, Glendora, CA, USA; TTR, Rocky Mountain Orthodontics, Denver, CO, USA; Idea, Leone, Sesto Fiorentino, Italy; 2D, Forestadent, Pforzheim, Germany) and a vestibular control bracket (Victory, 3M, Monrovia, CA, USA) were bonded according to manufacturers' instructions. A thin layer of primer (Transbond XT Adhesive, 3M, Glendora, USA) was applied to enamel surface with a microbrush; then the brackets were bonded with an adhesive resin (Transbond XT Resin, 3M, Glendora, USA) near the centre of the facial surface of the teeth. Excess adhesive was removed with a scaler and brackets were then light cured (Ortholux XT, 3M, Glendora, USA) for 20 seconds (5 seconds for each side of the bracket).

Half of the specimens (Groups 2, 4, 6, 8, and 10, coded as VI, STB, TTR, ID, and 2D, resp.) were submitted to UI (Mini Piezon, EMS, Nyon, Switzerland) with G1 tip (recommended by the manufacturer for supragingival scaling) and power setting of 8 W. The angulation between scaler tip and enamel was 0°, with the edge parallel to tooth surface [8]. UI was performed 15 seconds for each side of the bracket (total time: 1 minute). The water delivered directly from scaler unit was used as coolant. A single experienced and trained operator performed all the procedures.

The other half of the specimens did not receive any UI and served as control (Groups 1, 3, 5, 7, and 9, coded as VI + UI, STB + UI, TTR + UI, ID + UI, and 2D + UI resp.).

Subsequently all specimens were then secured in the lower jaw of an universal testing machine (Model 3343, Instron, Canton, MA, USA) and then tested in shear mode (head speed: 1 mm/min) [11]. The bonding surface of the brackets remained perpendicular to the horizontal plane and parallel to the direction of the force to be applied, in an effort to minimize peel and maximize shear during testing [8, 9, 13].

The maximum load necessary to debond bracket was recorded in newtons (N) and numeric values were converted into megapascals (MPa) as a ratio of newtons to surface area of the bracket.

The adhesive remnant index (ARI) score was recorded after specimen examination under optical microscope (Stereomicroscope SR, Zeiss, Oberkochen, Germany) at ×20 magnification, to assess the amount of adhesive left on the enamel surface [14]. ARI scale ranges from 0 to 3 (0: no resin remaining on tooth; 1: less than 50% resin remaining on tooth; 2: more than 50% resin remaining on tooth; 3: 100% resin remaining on tooth).

Statistical analysis was performed with software (R version 3.1.3, R Development Core Team, R Foundation for Statistical Computing, Wien, Austria). Descriptive statistics (mean, standard deviation, minimum, median, and maximum values) were calculated for all groups. The normality of the data was calculated using the Kolmogorov-Smirnov test. Analysis of variance (two-way ANOVA) and Tukey tests were applied for bond strength values. Linear regression was performed in order to evaluate the effect of bracket area on SBS values in control and UI groups. The chi-square test was used to determine significant differences in the ARI scores distributions among the different groups. Significance for all statistical tests was predetermined at *P* = 0.05.

## 3. Results

The descriptive statistics for the SBS (MPa) of the 10 groups tested are presented in Table 1. ANOVA showed the presence of significant differences (*P* < 0.0001). Post hoc Tukey test reported that, when evaluating control groups, significantly higher SBS values were reported with Groups 1 (Victory, vestibular), 5 (TTR, lingual), and 9 (2D, lingual). Significantly lower strengths (*P* < 0.05) were reported in Groups 3 (STB, lingual) and 7 (Idea, lingual).

UI significantly reduced SBS of some (TTR, Idea, and 2D) lingual brackets (Groups 6, 8, and 10) when compared with control groups (Groups 5, 7, and 9) (*P* < 0.05). On the other hand vestibular control bracket (Group 2, Victory) and a lingual bracket (Group 4, STB) showed no significant differences (*P* > 0.05) in SBS values when compared with their control groups (Groups 1 and 3, resp.).

**Figure 2:**
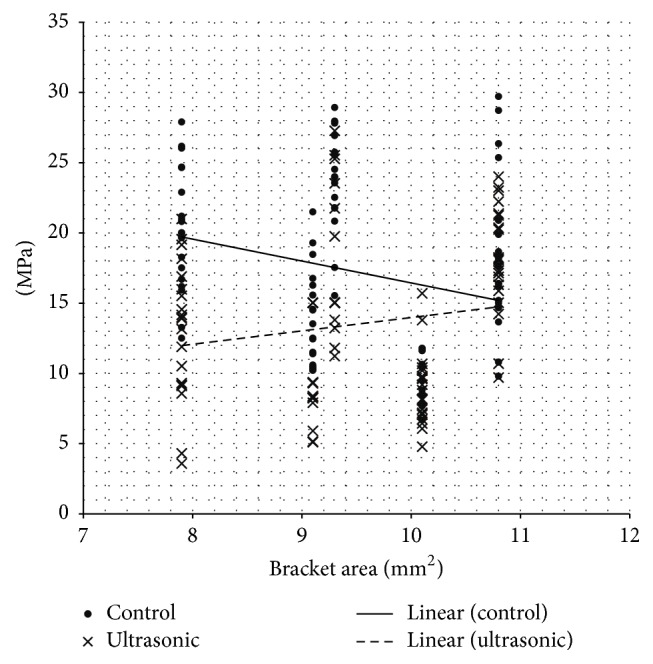
Linear regression of control and test (ultrasonic) groups.

Linear regressions (Figure 2) showed a significant effect of bracket base area on shear bond strength reduction after UI (*P* < 0.05). In fact, brackets with lower bracket base area (TTR, Idea, and 2D lingual brackets) showed significant decrease of SBS values after UI, whereas appliances with higher base surface (Victory vestibular brackets and STB lingual appliances) were not influenced by UI.

**Figure 3:**
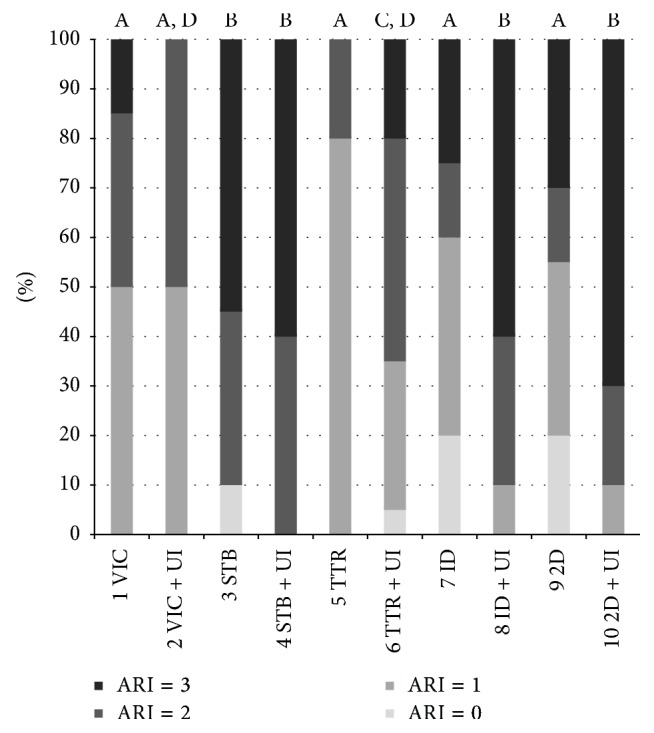
Frequency distribution of adhesive remnant index scores of the different tested groups.

When analysing ARI scores, Chi squared test showed significant differences among frequency distributions of various groups. For TTR, Idea, and 2D brackets a significant increase of ARI scores of “2” and “3” was reported after UI (*P* < 0.05). No significant differences in ARI scores distribution were reported for Victory and STB brackets (Figure 3) when comparing UI and control groups (*P* > 0.05).

## 4. Discussion

Then null hypothesis of the study has been rejected. After UI some lingual brackets (TTR, Idea, and 2D) showed SBS values significantly lower than control groups. On the other hand, a lingual bracket (STB) and the vestibular bracket (Victory) did not report significant differences between UI and control groups. As dental plaque is considered an important etiological factor in the development of caries and periodontal disease, the elimination of plaque and calculus is the prerequisite of all dental therapies. Ultrasonic instruments represent the principal treatment modality for these debris, as the vibration of scaler tips removes the deposits from the dental surface, such as bacterial plaque, calculus, and endotoxin [15]. The application of UI is widely used also in patients with prosthodontics rehabilitations, titanium implants, and orthodontic appliances [16]. The propagation of ultrasonic vibrations to dental implants has been reported to have physical effects on titanium surfaces, thus resulting in rougher external layer [17]. Moreover prolonged ultrasonic vibration at maximum power is used to facilitate the removal of posts, crowns, or bridges and could similarly debond orthodontic brackets [15]. Therefore UI is supposed to have significant effect on bonding materials due to the propagation of vibrations from the ultrasonic device to the resin, as well as to the biophysical action (cavitational activity and acoustic microstreaming) of ultrasound within the coolant (water) [8].

Therefore the application of UI has been postulated to have significant effects also on orthodontic brackets. In fact, a significant reduction of SBS values has been reported in brackets submitted to UI [8]. However, in this study, only a single vestibular bracket was considered. In the present report five different brackets were tested. When evaluating controls (no UI performed), Groups 1 (Victory, vestibular), 5 (TTR, lingual), and 9 (2D, lingual) showed the highest SBS values and no significant differences were reported among them. Significantly lower SBS were reported in Groups 3 (STB, lingual) and 7 (Idea, lingual). This baseline difference among the various braces is probably due to the differences in respective mesh anatomies. As showed in Figure 1, the various brackets showed significant variations of base form and size. In fact Groups 1, 2, 4, and 5 present bases with regular squared retentions, whereas Group 3 presents a mesh with irregular shape. Also among similar bracket bases (Groups 1, 2, 4, and 5) other differences can be detected, inherent to different dimensions of retention squares (smaller in Group 5 and bigger in Groups 1, 2, and 4). Moreover, from SEM images other differences can be found, such as sandblasted (Group 1) or not sandblasted (Groups 2, 3, 4, and 5) surfaces. Previous studies reported a significant effect of bracket mesh retention on SBS values for both vestibular [13, 18] and lingual [19] brackets. In fact the morphology of the base design may improve the penetration of the adhesive material [20] and this can explain the differences in SBS reported in the present investigation among untreated brackets (Groups 1 to 5). In order to enhance the retention of the adhesive to the metal base of orthodontic brackets, various chemical and mechanical retentive designs have been suggested [13]. Mechanical retention was enhanced by enlarging the size of the base, by placing undercuts in the cast bracket bases, by welding different diameter mesh wires to the bracket base, as well as incorporating different designs in the mesh itself [18]. In the present report, as showed in Figure 1, STB and Idea showed a conventional 80-gauge base, with retention wires parallel to bracket sides. These brackets showed the lowest SBS values when tested as control groups (no UI). Also Victory and 2D brackets showed the same conventional mesh, but with a 45° angulation between the retention wires and bracket sides. These braces showed higher bond strength values. On the other hand TTR bracket presented a different anatomy, with manufacturer name embedded in bracket base, thus resulting in a mesh with narrow and irregular grooves. These devices showed SBS similar to Victory and 2D. The morphology of the base design (and also the difference between vestibular and lingual bracket type) may alter the penetration of the adhesive material [13]. As in previous investigations that tested different devices [21, 22] the pad with narrower grooves and undercuts could have improved resin adhesion on a bracket base. Moreover in the present report brackets with a 45° angulation between retention wires and bracket sides showed higher SBS than bases with retention wires parallel to bracket sides. A possible explanation for the results could be that changing the surface pattern allows increased base roughness and microretentions and high bond strength [18].

After UI the reduction of SBS values recorded in our report was significantly different among the various groups. In fact, linear regressions showed a significant effect of bracket base area in lowering SBS values after UI. As showed, appliances with lower mesh area significantly reduced their adhesion capacity after UI, whereas brackets with higher mesh area did not reduce their SBS values after UI. Therefore, dental hygienists should pose particular care when applying ultrasonic vibrations around bracket bases, especially with brackets of small dimensions.

In the present investigation both lingual and vestibular brackets were bonded onto buccal surface. In fact lingual surface of bovine teeth has been reported to be more rough and curve than vestibular side, thus reducing repeatability of bond strength measurements. Therefore, in order to minimize errors and bias during tests execution, buccal side has been selected [9]. However, in the interpretation of the results, the use of bovine teeth has to be taken into consideration, as recorded SBS values could be slightly different from those reported with human enamel [9].

In literature a minimum bond strength of 6 to 8 MPa is reported to be adequate for most clinical orthodontic needs [23], but adhesion forces should not be too strong (over 40–50 MPa) in order to avoid enamel loss after debonding [24]. Therefore, the ideal orthodontic biomaterial should have bonding forces included in the interval of 5–50 MPa, even if these limits are mostly theoretical [9]. In the present report SBS values ranged from 3 MPa to 30 MPa and some specimens of brackets with lower base area after UI showed some values under this interval limits. Therefore, UI can lower SBS values under a clinically acceptable bond strength value, thus rising possibility of unwanted bracket failure.

Finally, in the present work ARI scores have been calculated for all groups. For TTR, Idea, and 2D (lower surface areas) brackets a significant increase of ARI scores of “2” and “3” was reported after UI, whereas no significant differences in ARI scores distribution were reported for Victory and STB (higher surface area) brackets when comparing UI and control groups. Previous Authors showed no significant effect of UI on ARI scores [8]. The difference of the results can be due to the fact that a single bracket has been tested, whereas in the present investigation five different bases were evaluated.

An ARI = 0 means a higher adhesion of bonding system, more to the bracket base than to the tooth, during the debonding process. In this case, it is claimed that less time is involved for adhesive removal from tooth surface [25]. In contrast, an ARI = 3 indicates failure between the bracket and adhesive, thus lowering risk of enamel fracture upon removal [26]. The results of the present investigation suggest that UI can be performed before final debonding of small-sized orthodontic brackets in order to raise ARI scores (thus presumably reducing enamel fracture risk). In the literature previous reports that evaluated ARI scores showed contradicting results. Both insignificant [20] and significant [22] effects of base design on ARI scores have been previously reported. This is probably due to the different materials and study design presented in the various investigations. In fact, evaluating the data of our study, ARI scores seem to be more influenced from UI and base area than from mesh form.

The results of the present report could be useful for both dental hygienists and orthodontists but should be confirmed with other studies, testing other scaler tips (Teflon-coated, plastic, or nonmetallic), different power outputs of ultrasonic units, and different adhesive systems.

## 5. Conclusions

The present investigation showed that appliances with lower mesh area significantly reduced their adhesion capacity after UI. The correlation between SBS reduction after UI and lower bracket areas has been reported to be linear.

Moreover UI significantly increased ARI scores of brackets with smaller bases.

## Figures and Tables

**Table 1:** Descriptive statistics of shear bond strength values (MPa) of the different groups.

Group	Code	Bracket	Manufacturer	Condition	Area (mm^2^)	Mean	SD	Min	Mdn	Max	Tukey^*∗*^
1	VIC	Victory	3M	Control	10.8	18.80	5.67	10.09	17.33	30.60	A
2	VIC + UI	Victory	3M	Ultrasonic	10.8	18.68	4.06	9.99	18.54	24.73	A
3	STB	STB	Ormco	Control	10.1	10.16	1.37	6.88	10.30	11.99	B, C, D
4	STB + UI	STB	Ormco	Ultrasonic	10.1	9.06	2.65	4.93	8.75	16.17	B, D
5	TTR	TTR	RMO	Control	7.9	20.55	4.28	12.88	20.44	28.73	A, E
6	TTR + UI	TTR	RMO	Ultrasonic	7.9	13.30	5.07	3.68	13.47	21.62	C
7	ID	Idea	Leone	Control	9.1	12.79	3.56	9.27	11.59	20.60	B, C
8	ID + UI	Idea	Leone	Ultrasonic	9.1	6.91	1.02	5.27	7.21	8.24	D
9	2D	2D	Forestadent	Control	9.3	23.80	3.44	16.00	24.72	29.79	E
10	2D + UI	2D	Forestadent	Ultrasonic	9.3	19.12	4.59	11.57	18.54	28.09	A

^*∗*^Post  hoc significance: groups with the same letter are not significantly different.
